# Long non-coding RNA HCG18 promotes gastric cancer progression by regulating miRNA-146a-5p/tumor necrosis factor receptor-associated factor 6 axis

**DOI:** 10.1080/21655979.2022.2034565

**Published:** 2022-03-04

**Authors:** Xianwu Yang, Run Liu

**Affiliations:** Department of Gastroenterology, Shijiazhuang People’s Hospital, Shijiazhuang City, P. R. China

**Keywords:** HCG18, TRAF6, miR-146a-5p, p65, gastric cancer, proliferation

## Abstract

Although long non-coding RNAs (lncRNAs) have been demonstrated to be dysregulated in gastric cancer (GC), the function of lncRNA HCG18 (HCG18) in GC is elusive. Therefore, the study was designed to evaluate the underlying mechanism of HCG18 in GC. HCG18 and microRNA 146a-5p (miR-146a-5p) levels in GC were evaluated by RT-qPCR. The effects of miR-146a-5p and HCG18 on GC cell function were examined using Transwell assay, colony formation, and CCK-8 assays. Tumor necrosis factor receptor-associated factor 6 (TRAF6) and p65 expression levels were detected by Western blot. HCG18 and miR-146a-5p target genes were identified using luciferase reporter and bioinformatics assays. HCG18 expression was increased in GC. HCG18 overexpression significantly increased GC cell proliferation, invasion, and migration. Furthermore, HCG18 overexpression inhibited miR-146a-5p and upregulated TRAF6 and p65 expression. Finally, miR-146a-5p/TRAF6 was found to be involved in the role of HCG18 in GC progression *in vivo*. Altogether, HCG18 promotes GC progression via the miR-146a-5p/TRAF6 axis and could be a GC treatment target.

## GRAPHICAL ABSTRACT

**Figure:**
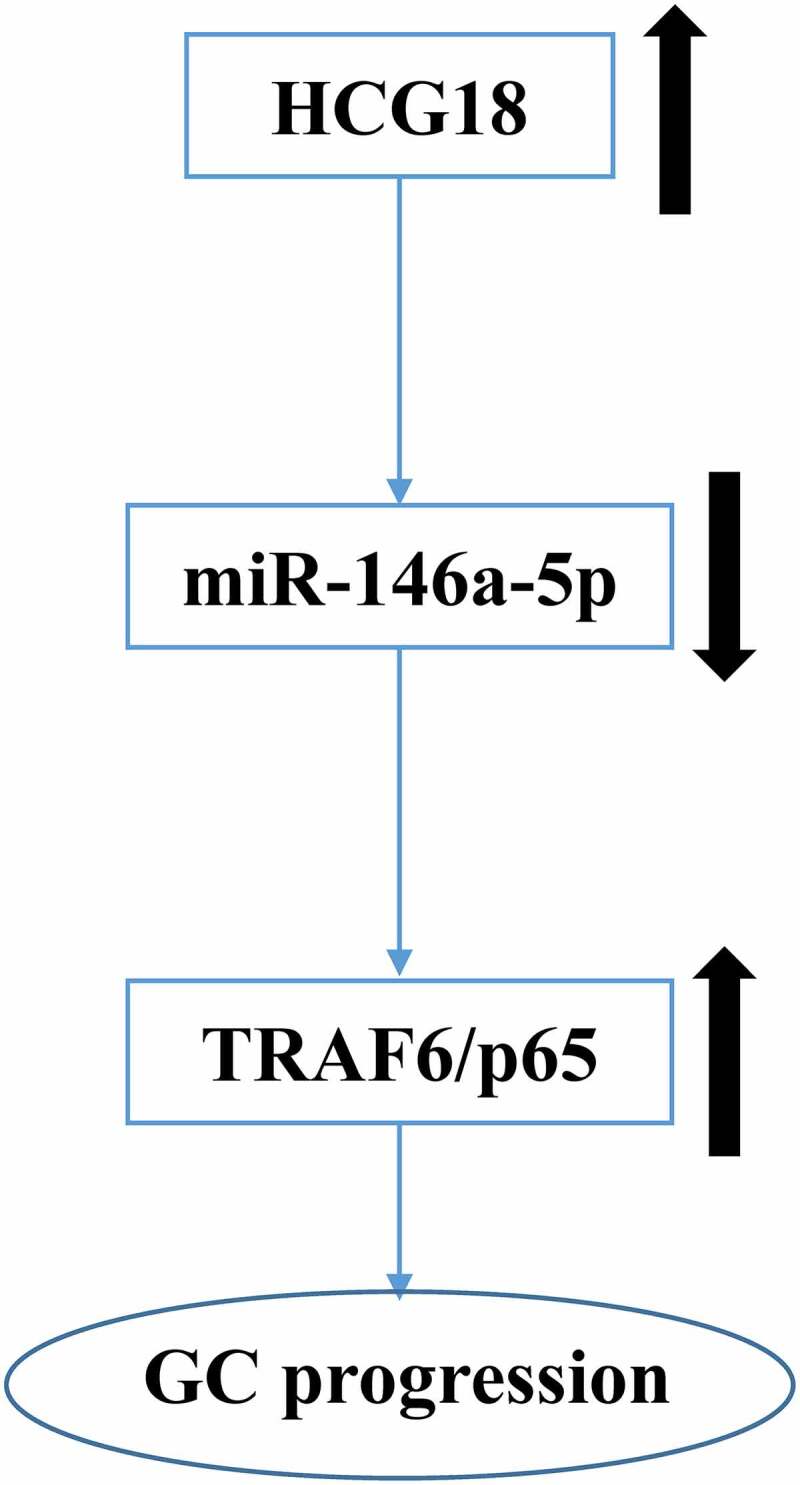

## Introduction

Gastric cancer (GC) is a digestive tract malignant tumor[1], [2] and has become one of the major diseases seriously endangering people’s health due to its annually increased incidence and decreased onset age [3]. The etiology and pathological mechanism of gastric cancer are complex, and environmental pollution, infection, and heredity are the main causes [4,5]. Most early gastric cancers have no obvious clinical symptoms, specific biomarkers, and effective treatments [6]. Molecular signal regulation network abnormalities are the molecular biological basis for GC development and the entry points for determining precise therapeutic targets and strategies [7]. Nowadays, studies have shown that non-coding RNAs (ncRNAs), including micro RNAs (miRNAs), long non-coding RNAs (lncRNAs), and circRNAs, affect GC cell proliferation, differentiation, and apoptosis [8,9].

LncRNAs are a class of ncRNA molecules [10,11] and participate in protein regulations [12]. LncRNAs can regulate cellular physiological functions at the genetic level by affecting genomic imprinting, chromatin packaging, cell differentiation, maintaining genome integrity, and embryonic development [13,14]. Abnormal expression of lncRNAs in tumors can promote tumor cell proliferation, invasion, and angiogenesis, inhibit tumor cell apoptosis, and affect tumor cell cycle progression, thereby promoting tumorigenesis and development [15,16]. Studies have reported that HCG18 is dysregulated in many tumors and can predict the prognosis of certain tumors [17]. It has been shown that HCG18 expression is abnormal in anaplastic glioma patients [18]. However, the role of HCG18 in GC has not been reported.

In recent years, it has been found that interactions between lncRNAs and miRNAs are involved in tumor development t [19]. As novel post-transcriptional regulators, miRNAs are closely related to life activities, such as disease occurrence, cellular metabolism, cell movement, apoptosis, differentiation and growth, and tissue and organ development [20]. Changes in miRNA expression in tumors are related to gene deletion, mutation, and polymorphism [21]. Some miRNAs have been shown to be associated with gastric cancer type, stage, and patient survival and may serve as molecular markers for tumor prognosis and diagnosis [22,23]. Many miRNAs are expressed in GC tissues and have received extensive attention in recent years [24]. For example, miR-133a-3p/FOXP3 axis regulates cell proliferation and autophagy in gastric cancer [25]. MKL1/miR-5100/CAAP1 loop regulates autophagy and apoptosis in gastric cancer cells [26]. Reduced miR-146a-5p expression is related to GC invasion, metastasis, venous vascular invasion, size, and differentiation and has clinicopathological significance in GC [27]. Clinical studies have found that tumor metastasis and invasion are closely related to the tumor necrosis factor receptor family [28]. LncRNAs may work through miRNA adsorption of downstream target genes [29]. Tumor necrosis factor receptor-associated factor 6 (TRAF6) specifically binds to certain protein ligands to block cell apoptosis [30]. P65 is a transcription regulator regulating cell proliferation and anti-apoptotic gene expression. TRAF6 and p65 are involved in GC development [31,32]. Therefore, we hypothesized that HCG18 might regulate GC progression via the miR-146a-5p/TRAF6 axis. The study aimed to investigate the mechanism of HCG18 in GC

## Methods

### Sample collection

According to the histopathological evaluation of patients diagnosed with GC, surgical resection was performed in Shijiazhuang People’s Hospital from 2013 to 2017. These patients were not treated locally or systemically before surgery. GC tissues and their adjacent normal tissues were collected from GC patients. The study was approved by the Ethics Committee of the Shijiazhuang People’s Hospital and conducted following the Declaration of Helsinki. All patients signed written informed consent and understood the experimental principle.

### Cell culture and transfection

Human gastric adenocarcinoma cell lines BSG823, HS-746 T, MKN-28, and 9811 and normal gastric cells (GSE1) were obtained from the Chinese Academy of Sciences Cell Bank (Shanghai, China) and cultured in DMEM (Weike, Shanghai, China) with 10% FBS. sh-HCG18 (5’-UUGGCUUCAGUCCUGUUCAUCAG-3’), sh-NC (5’-AAUUCUCCGAACGUGUCACGU-3’), sense miR-146a-5p mimic (5’-UGAGAACUGAAUUCCAUGGGUU-3’ antisense miR-146a-5p mimic (5’-AACCCAUGGAAUUCAGUUCUCA-3’), nonspecific mimic control (NC, sense 5’-UUUGUACUACACAAAAGUACUG-3’ and antisense 5’-CAGUACUUUUGUGUAGUACAAA-3’), inhibitor (sense 5’-AACCCAUGGAAUUCAGUUCUCA-3’) and NC (sense 5’-CAGUACUUUUGUGUAGUACAAA-3’) were obtained from Genepharma (Shanghai, China). SiRNA TRAF6 (5’-CCTGTGAATTTCAGAG-GCT-3’) and siRNA NC (5’-TTCTCCGAACGTGTCACGT-3’) were purchased from Santa Cruze (sc-36,717). Cells transfection was performed using RNAiMax and Lipofectamine 3000.

### Bioinformatic analysis

The putative miRNA binding sites on HCG18 and TRAF6 sequences were predicted using StarBase V3.0 (http://starbase.sysu.edu.cn/).

### Dua- luciferase reporter assay

Dual-luciferase reporter assay was performed as previously reported [33]. Human HCG18 was cloned into pGL3-basic vector (Promega). PGL3-REPOR-mutant (mut) vector was constructed by a pGL3-REPOR vector and a HCG18 fragment containing the putative miR-146a-5p binding target. After co-transfecting negative control with miR-146a-5p mimic, pGL3-REPOR-WT, or mut vector for 48 hours in MKN-28 cells, luciferase activity was measured.

### Quantitative real-time PCR (qRT-PCR)

Total RNAs were extracted using TRIzol reagent (Invitrogen, Carlsbad, CA, USA). Approximately 1 μg of total RNAs were reversely transcribed into cDNA using a Reverse Transcription Kit (Takara, Dalian, China), as previously described [34]. qRT-PCR assay was performed using the SYBR Green (Takara)-based ABI 7500 Detection System (Applied Biosystems, Foster City, CA, USA) with GAPDH and U6 as the internal references. The relative expression change of targets was analyzed using the 2^−ΔΔCt^ method. PCR conditions were 1 min at 95 °C followed by 40 cycles of 5 s at 95 °C and 34s at 60 °C. Three technical replicates were set for each experiment. The primers were HCG18 forward 5’-ATCCTGCCAATAGATGCTGCTCAC-3’ and reverse 5’-AGCCACCTTGGTCTCCAGTCTC-3’, GAPDH forward 5’-TGACGTGCCGCCTGGAGAAAC-3’ and reverse 5’-CCGGCATCGAAGGTGGAAGAG-3’, miR-146a-5p forward 5’-CGCGTGAGAACTGAATTCCA-3’ and reverse 5’-AGTGCAGGGTCCGAGGTATT-3’, TRAF6 forward 5’-GCCCATGCCGTATGAAGAGA-3’ and reverse 5’-ACTGAATGTGCAGGGGACTG-3’, and U6 forward 5ʹ-GAGGGCCTATTTCCCATGATT-3’ and reverse 502B9-TAATTAGAATTAATTTGACT-3’.

### Cell proliferation assay

MKN-28 cells at the log phase were collected and prepared as single-cell suspension. Cells were placed in 96-well plates with 10^3^ to 10^4^ per well and cultured for 24 hours. After adding CCK8 solution, the absorbance at 450 nm was determined using a microplate reader (PT-3502; Potenov, Beijing, China).

### Colony formation test

After forming a single dispersed cell suspension, transfected MKN-28 cells were placed in an incubator for 7 days and subjected to Wright’s staining for 10 min using a mixture of Sorensen phosphomolybdic acid buffer and Giemsa dye solution at a ratio of 9:1. After washing, colonies containing > 50 cells were counted under a microscope in 20 randomly selected fields.

### Transwell test

Migration and invasion assays were performed using 8-μm (pore size) Transwell chambers (Costar, Corning Inc., NY, USA) coated without or with Matrigel (BD Biosciences) as previously described [35]. In detail, invasion assays were conducted similar to migration assays, except the membranes of the upper chambers were pre-coated with Matrigel (30 μg/well). For migration assay, transfected MKN-28 cells were re-suspended in 200 µL of serum-free RPMI-1640 medium and seeded in the upper chambers. After 24 h of incubation, cells migrated or invaded through the membranes to the lower surface were fixed with ethanol, stained with 0.2% crystal violet, and observed and counted in five random fields using a microscope.

*In vivo* experiments

*In vivo* experiments were performed as previously reported [36]. Athymic BALB/c nude mice (Fifteen, female) were randomly divided into three groups (n = 5 per group). 2 × 10^7^ MKN-28 cells containing sh-HCG18, si-NC, or sh-HCG18 and anti-miR-146a-5p were injected subcutaneously into each mouse flank to construct the mouse xenograft model. The tumor size was measured twice a week. After 20 days, mice were sacrificed, and the tumors were collected and subjected to Western blot and qPCR analyses to detect genes of interest at RNA and protein levels. All experiments were approved by the Ethics Committee of the Shijiazhuang People’s Hospital and conducted strictly following the Shijiazhuang People’s Hospital guidelines for the use and care of experimental animals.

### Western blot

Total proteins were extracted and quantified by BCA Protein Assay Kit and subjected to Western blot analyses as detailed previously [37] using primary antibodies against GAPDH (1:1000), TRAF6 (1:500), p-P65 (1:500), and P65 (1:500) and anti-rabbit secondary antibody (1:5000) from Proteintech, Chicago, USA.

### Immunohistochemistry

Tissue samples were fixed in 10% formalin (10%) and prepared as 4 μm thick paraffin sections. These paraffin sections were subjected to heat-induced epitope repair, incubated with human Ki-67 (1:200, Abcam), and observed under a light microscope by two pathologists to assess proteins’ cellular localization and immunostaining levels.

### Statistical Methods

Data were analyzed by SPSS19.0 and presented as mean ± standard deviation (SD). Differences among multiple groups were analyzed using one-way ANOVA and followed LSD test. P < 0.05 indicated a statistical significance.

## Results

This study aimed to investigate whether and how HCG18 is involved in GC progression. We found that HCG18 promoted GC progression via the miR-146a-5p/TRAF6 axis and might be a target for GC treatment.

### HCG18 in GC tumorigenesis

**Figure 1.**
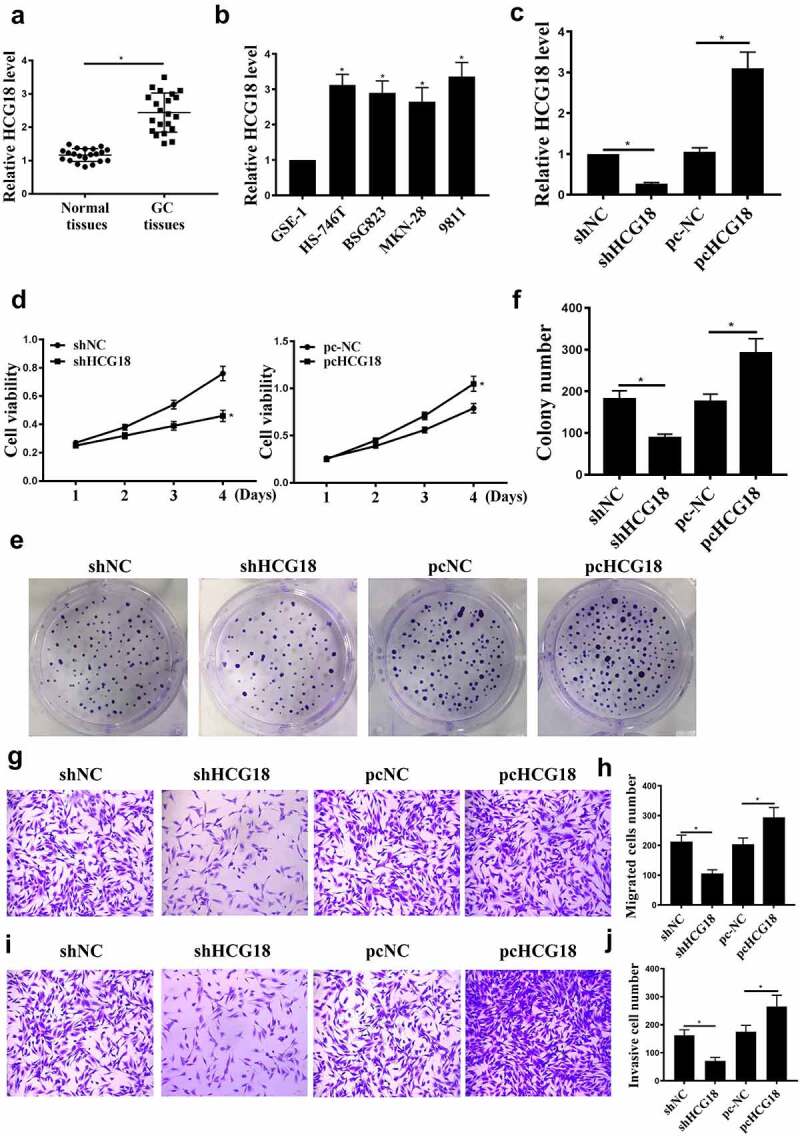
The role of HCG18 in GC. (a) HCG18 mRNA levels in GC tumor and normal tissues (n = 21) were detected by qRT-PCR. (b) HCG18 mRNA levels in BSG823, HS-746 T, MKN-28, and 9811 cell lines were detected by qRT-PCR. (c) Under different treatment conditions, HCG18 mRNA levels in MKN-28 cells were detected by qRT-PCR. (d) MKN-28 cell proliferation was detected by CCK-8 assay. (e and f) MKN-28 cell proliferation was detected by colony formation assay. (g-j) MKN-28 cell invasion and migration were detected by transwell assay. n = 3, * p < 0.05.

For analyzing HCG18 function in GC, HCG18 expression in 21 GC tissues was examined. HCG18 expression was increased in GC tissues than in adjacent normal tissues (Figure 1(a), p < 0.05) and significantly upregulated in human gastric adenocarcinoma cell lines (BSG823, HS-746 T, MKN-28, and 9811) compared with GSE-1 (Figure 1(b), p < 0.05). In addition, HCG18 expression was not significantly different among the 4 cell lines. Therefore, MKN-28 was chosen in subsequent experiments.

MKN-28 cells were transfected with sh-HCG18, sh-NC, pc-NC, or pc-HCG18. As shown in Figure 1(c), HCG18 upregulation and downregulation were observed, indicating successful transfection. HCG18 knockdown inhibited MKN-28 cell proliferation (Figure 1(d), p < 0.05), migration, and invasion (Figure 1(g), p < 0.05). In addition, HCG18 overexpression promoted MKN-28 cell proliferation, migration, and invasion Figures 1(f), 1 J, p < 0.05). These data indicate that HCG18 is involved in GC proliferation and metastasis.

### HCG18 targets miR-146a-5p

**Figure 2.**
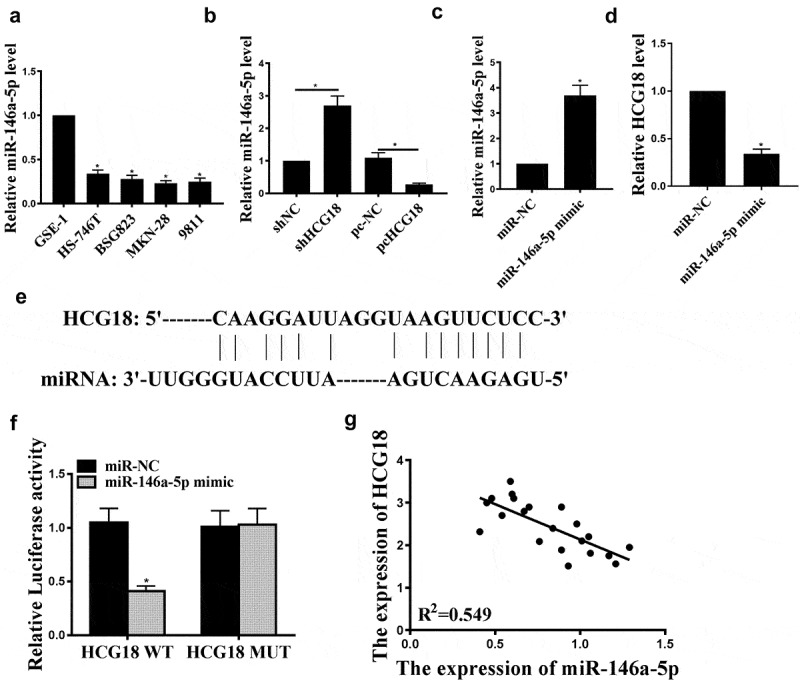
HCG18 regulated miR146a-5p expression in GC cells. (a) miR146a-5p mRNA levels in BSG823, HS-746 T, MKN-28, and 9811 cell lines were detected by qRT-PCR. (b-c) MiR-146a-5p levels in MKN-28 cells under different treatment conditions were evaluated by qRT-PCR. (d) HCG18 mRNA level in cells with miR-146a-5p overexpression was detected by qRT-PCR. (e) The putative target sequence between HCG18 and miR146a-5p was predicted using StarBase V3.0 (http://starbase.sysu.edu.cn/). f. Luciferase reporter assay was performed in MKN-28 cells. g. Correlation between miR-146a-5p and HCG18 in GC tissues was evaluated by Pearson’s correlation analysis. n = 3, * p < 0.05.

Next, we examined miR-146a-5p expression in human gastric adenocarcinoma cell lines (BSG823, HS-746 T, MKN-28, and 9811) and found that miR-146a-5p was significantly reduced in human gastric adenocarcinoma cell lines than in normal gastric cell line GSE-1 (Figure 2(a), p < 0.05). Furthermore, HCG18 knockdown increased miR-146a-5p expression in MKN-28 cells, and HCG18 overexpression reduced miR-146a-5p expression (P < 0.05, Figure 2(b)). MiR-146a-5p transfection successfully increased miR-146a-5p level (Figure 2(c), p < 0.05) and decreased HCG18 expression (Figure 2(d), p < 0.05). Furthermore, StarBase v. 2.0 predicted that miR-146a-5p might be a potential target of HCG18 (Figure 2(e)). Luciferase activity was reduced in cells transfected with pGL3-REPOR-HCG18-WT but not in cells transfected with pGL3-REPOR-HCG18-mut (Figure 2(f)). HCG18 negatively correlated with miR-146a-5p (Figure 2(g)). Altogether, miR-146a-5p may mediate HCG18 function in GC.

### HCG18 function is mediated by miR-146a-5p in GC

**Figure 3.**
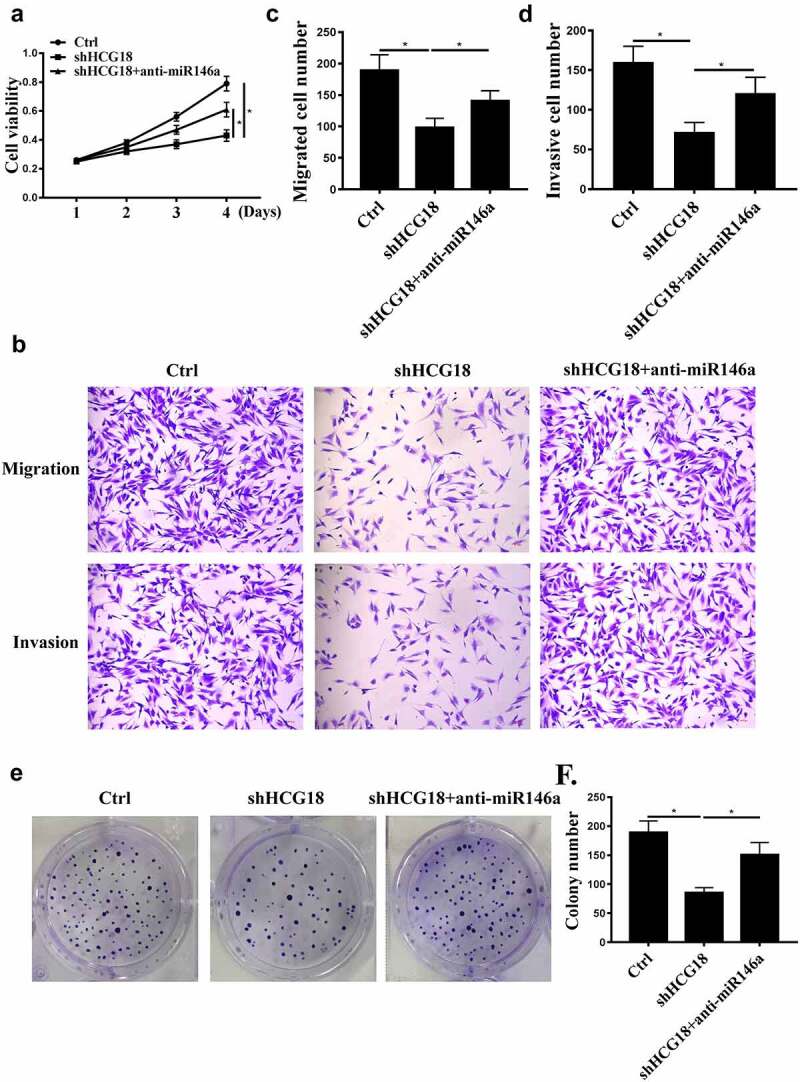
The effects of HCG18 on GC cells were mediated by miR-146a-5p. (a) MKN-28 cell viability was detected by CCK-8 assay. (b-d) MKN-28 cell invasion and migration were evaluated using Transwell assays. (e and f) MKN-28 cell proliferation was detected by colony formation. n = 3, * p < 0.05.

MiR-146a-5p inhibitor and shHCG18 were used to further analyze the underlying mechanism of HCG18 in GC. We found that HCG18 knockdown inhibited MKN-28 cell proliferation, migration, and invasion (Figure 3(a-c), P < 0.01), and these effects were partially attenuated by miR-146a-5p inhibition (Figures 3(d-f)). These results suggest that miR-146a-5p mediates the function of HCG18 in GC.

### TRAF6 is a direct target of miR-146a-5p

**Figure 4.**
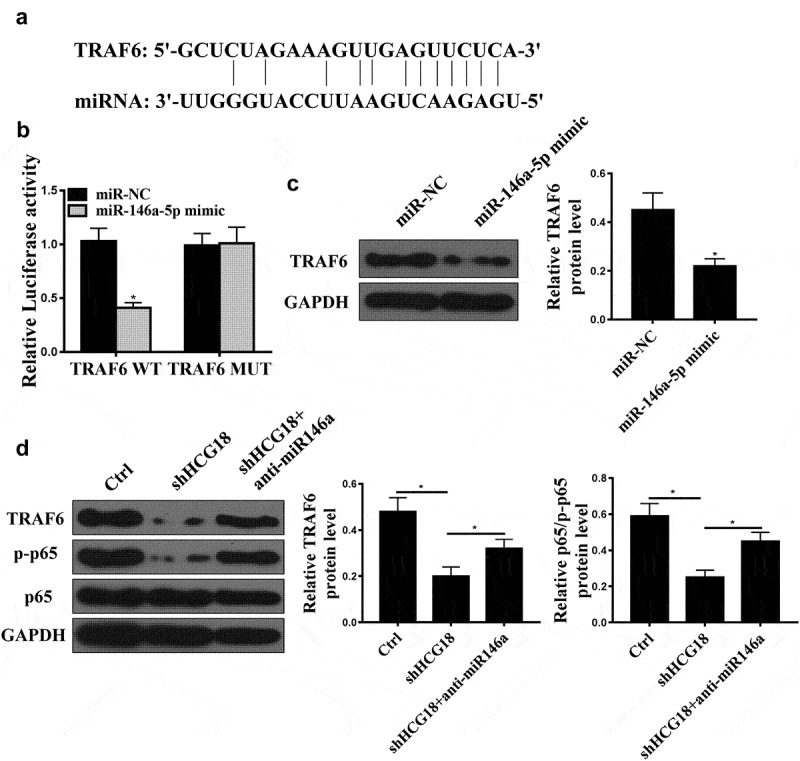
TRAF6 was a direct target of miR-146a-5p. (a) Target sequence of miR-146a-5p on the 3’-UTR of TRAF6 was predicted using StarBase V3.0 (http://starbase.sysu.edu.cn). (b) Luciferase activity was measured in MKN-28 cells. (C) TRAF6 protein level in MKN-28 cells after miR-14a-5p mimic transfection was measured by Western blot. (d) TRAF6 and p65 protein levels in MKN-28 cells were detected using Western blot. * P < 0.05, n = 3.

Bioinformatics analysis predicted that TRAF6 might be a target of miR-146a-5p (Figure 4(a)). In addition, we found that miR-146a-5p mimic reduced the luciferase activity of cells transfected with pGL3-REPOR-TRAF6-WT but not cells transfected with pGL3-REPOR-TRAF6-mut (Figure 4(b)). Moreover, miR-146a-5p overexpression reduced TRAF6 protein expression in GC cells (Figure 4(c), p < 0.01). Furthermore, shHCG18 reduced TRAF6 and p-p65 expression, while co-transfection of anti-miR-146a-5p reversed the effect of HCG18 on TRAF6 and p-p65 expression (Figure 4(d)).

### TRAF6 downregulation attenuates the effects of miR-146a-5p on GC

**Figure 5.**
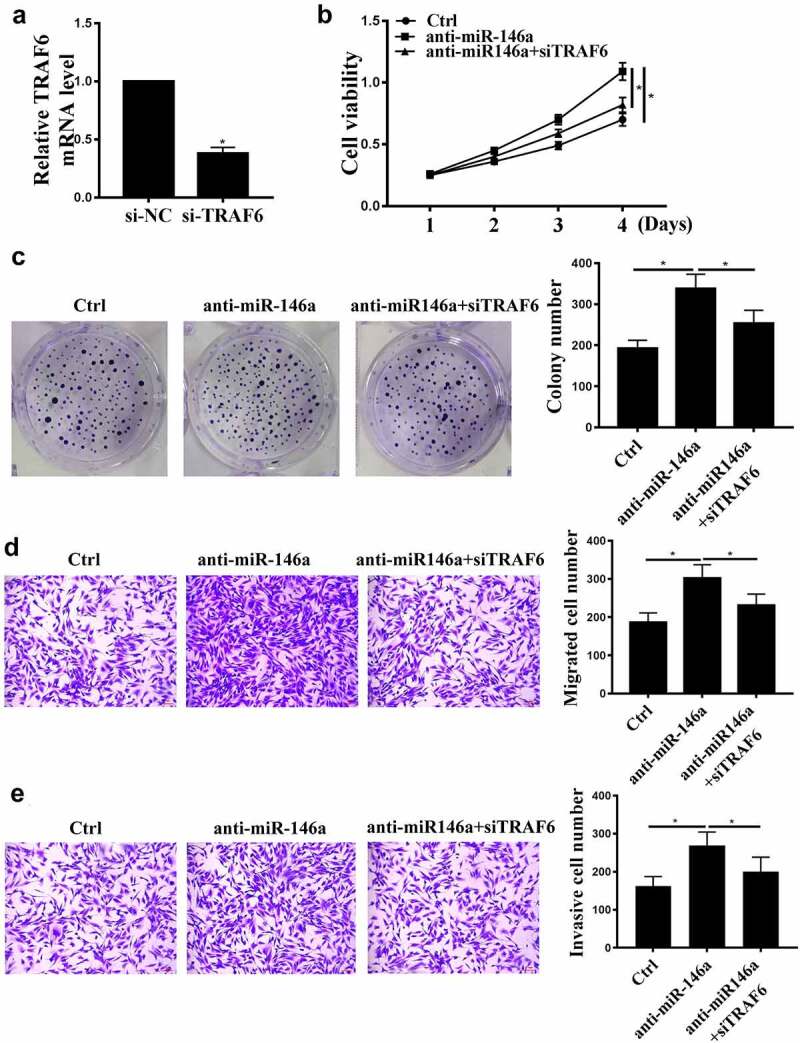
The effects of TRAF6 on GC. (a) TRAF6 mRNA levels after si-TRAF6 transfection were detected by qRT-PCR. (b) MKN-28 cell viability was detected by CCK-8 assay. (c) MKN-28 cell proliferation was detected by colony formation assay. (d), (e) MKN-28 cell invasion and migration were detected by Transwell assay. n = 3, * p < 0.05.

We further carried out rescue experiments. si-TRAF6 transfection reduced TRAF6 expression compared with control siRNA (Figure 5(a), p < 0.05), indicating successful transfection. MiR-146a-5p inhibition induced cell proliferation, invasion, and migration (Figure 5(b-e)), and these effects were partially attenuated by TRAF6 knockdown (P < 0.01, Figure 5(b-e), P < 0.01).

### HCG18 promotes GC tumor growth in vivo

**Figure 6.**
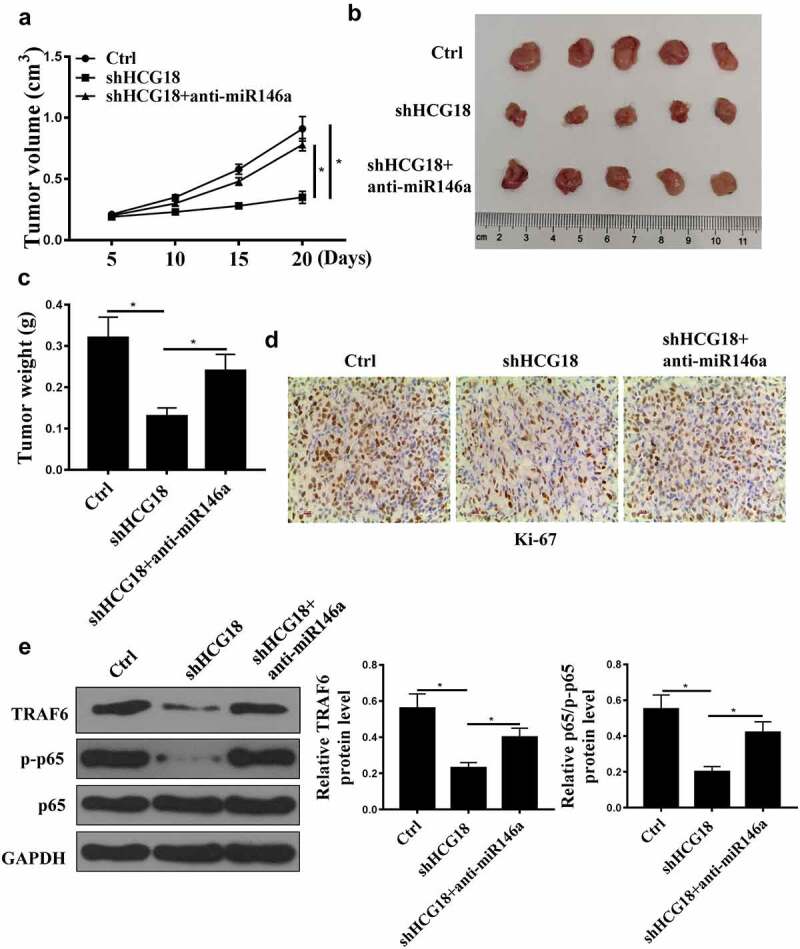
HCG18 promoted tumor growth *in vivo*. (a) GC tumor volume in different groups was detected. (B) Tumor tissues in different groups were photographed. (c) GC tumor weight in different groups was measured. (d) Immunochemistry of tumor tissues in different groups was detected using Ki67 assay. (E) TRAF6 and p65 protein levels were detected by Western blot. * p < 0.05.

HCG18 knockdown reduced tumor volume and weight, and miR-146a-5p inhibition reversed the effect of HCG18 knockdown on tumor weight and volume (Figures 6(a-c), P < 0.01). In addition, HCG18 knockdown reduced Ki67-positive cell number, while miR-146a-5p inhibition reversed the effect of HCG18 knockdown on Ki67-positive Pcells (Figure 6(d)). Furthermore, TRAF6 and p-p65 protein levels were decreased by HCG18 knockdown and elevated by anti-miR-146a-5p transfection (Figure 6(e), p < 0.01). These data demonstrate that HCG18 may promote GC tumor growth via miR-146a-5p/TRAF6.

## Discussion

GC is one of the most common malignant tumors worldwide [38]. However, due to the concealment nature of GC, the proportion of patients with early GC is very low, and high-specificity and high-sensitivity molecular markers for effective GC diagnosis have not been identified [2,39,40]. GC has become a public health problem to be solved urgently.

Chemotherapy is still an important means of GC treatment. However, chemotherapeutic drugs cannot accurately distinguish normal cells and tumor cells due to lack of an effective chemotherapeutic window and side effects [41,42]. Currently, molecular targeted therapy has received wide attention because of its clear target, definite curative effect, low toxic and side effects, and good patient tolerance [43]. LncRNAs play a critical role in many life activities [44]. There is increasing evidence that lncRNA involves in monitoring and evaluation of gastric cancer prognosis [45]. Some research groups have found that lncRNACCAT2 and lncRNA CARLo.5 are elevated in GC tissues. High levels of lncRNACCAT2 and lncRNACARLo-5 are significantly associated with advanced T stage and distal metastasis of gastric cancer patients, and the overall survival of patients Period and disease-free survival are poor [46,47]. Moreover, lncRNA00265 was also found to promote GC cell proliferation [48]. HCG18 plays a regulatory role in multiple tumors [49]. In this study, we found that HCG18 expression in GC was increased, and HCG18 knockdown inhibited GC cell function, while HCG18 overexpression had opposite results. In addition, tumor growth was inhibited by HCG18 knockdown. These results suggest that HCG18 may be an oncogene in GC development.

LncRNAs can directly bind to target mRNAs to regulate their expression via miRNAs [50]. Studies have shown that lncRNA HOTAIR upregulation in GC may promote human epidermal HER2 cell invasion by binding to miR-331-3p, thereby promoting gastric cancer cell invasion [51]. LINC00265 promotes GC cell proliferation via the miR-144-3p/CBX4 axis [48]. In addition, it has been found that lncRNA TUSC7 inhibits miR-23b to target genes [52]. MiR-146a-5p is differentially expressed in many diseases [53]. Here, we found that miR-146a-5p expression was reduced in GC cells and was negatively regulated by HCG18. MiR-146a-5p inhibition partially eliminated the effect of HCG18 knockdown on GC cell function. These indicate that miR-146a-5p mediates HCG18 function in GC. Our study was consistent with a previous report that HCG18 promotes intervertebral disc degeneration by sponging miR-146a-5p and regulating TRAF6 expression [54]. Therefore, whether TRAF6 also mediates the roles of HCG18 and miR-146a-5p in GC needs to be further evaluated.

It has been reported that interactions between miRNAs and lncRNAs affect tumor occurrence and development [55]. TRAF, as a ligand protein, plays a number of important biological roles, such as regulating cell survival, proliferation, and apoptosis and inducing endothelial cell differentiation [56]. TRAF6 is also involved in tumor development, invasion, and metastasis [57]. Recently studies have found that TRAF6 and p-p65 promote GC cell invasion and metastasis and predict a poor prognosis [31,58]. This study found that miR-146a-5p regulated TRAF6 expression, and anti-miR-146a-5p reversed the effect of shHCG18 on TRAF6 and p-p65 protein levels. Anti-miR-146a-5p induced GC cell migration and proliferation and these effects were partially attenuated by si-TRAF6. These data indicate that HCG18 regulates TRAF6 by sponging miR-146a-5p in GC (graphical abstract).

## Authors’ contributions

XWY: experiment studies, statistical analysis, literature search and data analysis; RL: research design, manuscript writing, manuscript editing and project administration. All authors read and approved the final manuscript.

## Availability of data and material

The datasets used and/or analyzed during the current study are available from the corresponding author on reasonable request.

## Conclusion

Our results indicated that HCG18 acts as a ceRNA to regulate TRAF6 by sponging miR-146a-5p in GC and may be a target for GC treatment.
